# Technical Report: Machine-Learning Pipeline for Medical Research and Quality-Improvement Initiatives

**DOI:** 10.7759/cureus.46549

**Published:** 2023-10-05

**Authors:** Alexander A Huang, Samuel Y Huang

**Affiliations:** 1 Surgery, Northwestern University Feinberg School of Medicine, Chicago, USA; 2 Internal Medicine, Icahn School of Medicine at Mount Sinai South Nassau, Oceanside, USA

**Keywords:** data analytics, medical simulation, xgboost, shapely additive explanation, ai and machine learning

## Abstract

Machine-learning techniques have been increasing in popularity within medicine during the past decade. However, these computational techniques are not presented in statistical lectures throughout medical school and are perceived to have a high barrier to entry. The objective is to develop a concise pipeline with publicly available data to decrease the learning time towards using machine learning for medical research and quality-improvement initiatives. This report utilized a publicly available machine-learning data package in R (MLDataR) and computational packages (XGBoost) to highlight techniques for machine-learning model development and visualization with SHaply Additive exPlanations (SHAP). A simple six-step process along with example code was constructed to build and visualize machine-learning models. A concrete set of three steps was developed to help with interpretation. Further teaching of these methods could benefit researchers by providing alternative methods for data analysis in medical studies. These could help researchers without computational experience to get a feel for machine learning to better understand the literature and technique.

## Introduction

Machine learning shows promise for the development of prediction models and clinical prediction rules that could improve care in the medical field [1,2]. Proponents of the use of machine learning in healthcare claim that these systems enhance prognostic accuracy and can be adapted towards benefiting patients with more accurate diagnosis, prognosis, and personalized therapeutics [3,4].

Despite the potential benefits of machine learning, these models are complex in their interpretation and come with multiple potentials for incorrect use [5,6]. First, these methods are often difficult to implement, requiring multistep data processing methods as well as the construction of training and testing sets. Second, these methods are often cryptic [7,8]. They are colloquially referred to as a “black box”, in which these algorithms may generate a prediction but provide little information regarding how the result was achieved [9]. Thus, researchers may not know whether a particular signal from an important covariate was helping in prognosis, or if demographic factors led to the prediction and are replicating previous biases of the healthcare system [10]. Third, the evaluation of these algorithms does not have the traditional “p-value” output for each covariate that was common in most methods used in the medical literature (e.g., linear regression, survival analysis, logistic regression) [11,12].

Given the limitations of the literature on machine-learning methods, we aim to develop a simple six-step process for model building and three recommendations for interpretation of the model output that will help provide a sense of machine-learning use in medicine.

## Technical report

Materials

One of the most commonly used software for data analysis in medicine is R and its environment R Studio. Thus, the free version of R (https://cran.r-project.org/) and the free version of R-studio (https://www.rstudio.com/products/rstudio/download/) are used in this paper.

Methods

The five-step plan will be presented in the following format: a heading detailing the goal of each step, a rationale component for the explanation of the usefulness of the step, the execution, detailed step-by-step instructions that can be replicated, and the code, r-code that can be pasted into the software and run by the reader.

The five-step data fitting plan

Step 1: Installation of Libraries

Rationale: Machine-learning techniques have been studied for decades by numerous pioneers in statistics and computer science. They often require numerous optimization steps and their development requires an intricate understanding of the differential equations and statistical computation. However, decades of research have been compiled into free, simple-to-use functions that can be implemented by the user.

Execution: Installation of numerous libraries: MLDataR for the main data, and farff, dplyr, xgboost, ggplot2, SHAPforxgboost for data-cleaning, model fitting, and model visualization.

Code: *install.packages(Package Name) (e.g., install.packages("MLDataR"))*

Step 2: Extracting and Cleaning Data

Rationale: Before any modeling, it is necessary to pick out the needed variables that will be used in the model and to make sure that the data is free from errors.

Execution: Extraction of the heart disease cohort and selection of covariates into a vector.

Code:

data(heartdisease)

df <- heartdisease

x = c("Age" , "Sex" , "RestingBP" , "Cholesterol" , "FastingBS" ,"RestingECG" , "MaxHR", "Angina", "HeartPeakReading")

Step 3: Split Into Training and Testing Sets

Rationale: It is important to split the data into two sets, 80% of which going to training and 20% going to testing.

This split can be understood through traditional learning. If a student is studying for an exam, they may use 70% of the practice questions to learn, checking the answers to get a better understanding of the material. After learning from these 70% of the questions, the student would then need a “test” to be able to truly assess whether they learned or not. They are evaluated based on their knowledge of the remaining 30% of questions that were not previously presented to them.

Just like it would not be accurate to allow a student to study on questions that are on the test themselves, we cannot let a model be fitted on data that will be used in its validation.

Execution: Randomly sample 80% into a training set and 20% into a testing set. Note, these ratios can be changed depending on the size and application of the data.

Code:

ix <- sample(nrow(df), 0.8 * nrow(df))

dtrain <- xgb.DMatrix(data.matrix(df[ix, x]), label = df[ix, ]$HeartDisease)

dvalid <- xgb.DMatrix(data.matrix(df[-ix, x]), label = df[-ix, ]$HeartDisease)

Step 4: Parameter Selection and Model Fitting

Rationale: After cleaning data and splitting it into train and test, it is finally time to fit the model. Select parameters based on the literature around the data. Methods to select parameters may vary from field to field. Example parameters given.

Execution: 1) Set the desired parameters and 2) fit the model. 

Code:

params <- list(

 objective = "reg:squarederror",

 learning_rate = 0.05,

 subsample = 0.9,

 colsample_bynode = 1,

 reg_lambda = 2,

 max_depth = 5

)

fit_xgb <- xgb.train(

 params,

 data = dtrain,

 watchlist = list(valid = dvalid),

 early_stopping_rounds = 20,

 print_every_n = 100,

 nrounds = 10000 # early stopping

)

Step 5: Visualization of the Data

Rationale: Machine-learning algorithms do not present clear slopes for each covariate like linear regression or hazard ratios, like survival analysis. However, we can utilize Shapely Additive Explanations (SHAP), to visualize each covariate in the model.

Execution: Visualize each covariate in the plot in R studio.

Code:

X <- data.matrix(df[, x])

shap <- shap.prep(fit_xgb, X_train = X)

shap.plot.summary(shap)

for (v in shap.importance(shap, names_only = TRUE)) {

 p <- shap.plot.dependence(shap, v, smooth = TRUE,

 alpha = 0.5, jitter_width = 0.1) +

 ggtitle(v)

 print(p)

}

Three steps for model evaluation and interpretation

Step 1: Model Statistics

Use of the code: “pred <- predict(fit_xgb, dvalid)” can be used to extract the model predictions. Additionally, “df[-ix, ]$HeartDisease” can be used to extract the actual results. Comparisons between actual and predicted results can be compared through these two vectors that have already been coded. Different fields require different model results so these will not be covered in this paper; however, some common features include sensitivity, specificity, positive likelihood ratio, negative likelihood ratio, and Area under the Receiver Operator Characteristic curve. There are multiple R-packages that will help the computation of these.

Step 2: Visualization of Model Output From SHAP

**Figure 1:**
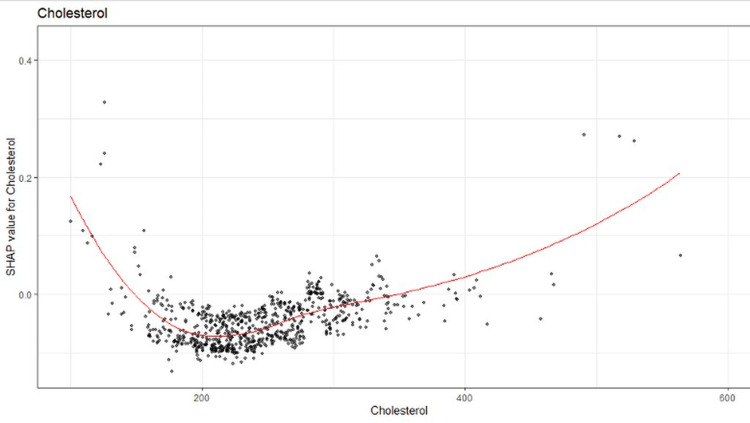
SHAP visualization of the relationship between cholesterol and heart disease The SHAP plot shows the nonlinear relationship between cholesterol and heart disease. Note that these data are used to demonstrate SHAP visualization and machine learning, not for the study of the relationship between cholesterol and heart disease.

SHAP allows for the visualization of each covariate. One example covariate is cholesterol. From the model, we were able to extract the non-linear relationship between cholesterol and mortality and we can interpret this visualization as seen in Figure 1.

Step 3: A Word of Caution in Using Machine Learning

Machine learning provides many benefits: including having a model without the traditional assumptions of linearity (in the case of linear models) or proportional hazards (in the case of cox proportional hazards models for survival analysis). It often outperforms traditional parametric models (linear regression, logistic regression) as well. However, machine-learning algorithms are still cryptic. It is impossible for humans to compute by hand the calculations made by a computer, much less understand each calculation made. Thus, caution must be made that the model is not overfitting (we can partially account for this by making sure the prediction accuracy for the testing set is not much worse than the training set), and by visualizing the results from SHAP (to make sure that the covariates that we place into the model match our understanding (e.g., the cholesterol plot visualization). Additionally, traditional parametric models should be used as a front line to understand the data if the plan is to use more involved methods.

## Discussion

The presented technical report introduces a streamlined machine-learning pipeline designed to bridge the gap between medical professionals and the complexities of machine learning. This section discusses the significance of this pipeline in the context of medical research and quality-improvement initiatives, addresses potential challenges and limitations, and explores the broader implications of integrating machine learning into medical education.

The adoption of machine-learning techniques in medical research has the potential to revolutionize clinical decision-making and patient care. The pipeline outlined in this report offers medical professionals an accessible entry point into this realm. By providing step-by-step guidance, the pipeline facilitates the creation of predictive models using real-world medical data. This empowers researchers to uncover hidden patterns, relationships, and trends in patient data, contributing to more accurate diagnoses, treatment plans, and overall healthcare outcomes [13]. Machine learning's ability to extract valuable insights from complex medical data can greatly benefit quality-improvement initiatives within healthcare institutions. The outlined pipeline equips healthcare practitioners with the tools needed to analyze historical data, identify areas for improvement, and implement data-driven interventions. This could lead to enhanced patient safety, optimized workflows, and ultimately improved healthcare delivery. Machine-learning techniques are often perceived as technically daunting, which can discourage medical professionals from exploring their potential [14]. The presented pipeline alleviates this barrier by providing clear explanations, practical examples, and ready-to-use code. This approach empowers individuals with limited computational experience to harness the power of machine learning, fostering a culture of innovation and continuous learning within the medical community.

Challenges and limitations

While the pipeline streamlines the process of using machine learning, challenges remain. Medical professionals must still possess a solid understanding of the underlying principles to appropriately interpret and apply the results. The pipeline's simplicity may inadvertently downplay the complexities inherent in model development, leading to oversimplified assumptions. Additionally, the pipeline assumes access to high-quality and well-curated medical data, which may not always be readily available [15].

Broader implications for medical education

The integration of machine learning into medical education is critical for preparing the next generation of healthcare professionals. Incorporating computational techniques early in medical curricula could help mitigate the perception of machine learning as an exclusive domain. By equipping medical students with foundational knowledge and practical skills, educators can ensure that future healthcare providers are well-prepared to leverage advanced technologies for improved patient care.

Future directions

Expanding on the presented pipeline, future research could focus on refining the process and incorporating more advanced machine-learning algorithms. Additionally, incorporating interdisciplinary collaboration between medical professionals, data scientists, and machine-learning experts could lead to the development of more sophisticated models that address nuanced medical challenges.

## Conclusions

Machine learning provides a multitude of benefits to medicine. It allows for leveraging the advances in processing power, memory, storage, and data to develop more accurate clinical predictions. This guide is designed to allow individuals with limited computational experience to experience the process of building and understanding a machine-learning model. Users should contemplate the benefits and detriments to the utilization of these models and make careful choices as this will help new machine-learning users to fit their first model and may help experienced researchers acquire a streamlined platform for using these algorithms.
